# Detection of SARS-CoV-2 variant 501Y.V2 in Comoros Islands in January 2021

**DOI:** 10.12688/wellcomeopenres.16889.1

**Published:** 2021-07-28

**Authors:** Charles N. Agoti, George Githinji, Khadija S. Mohammed, Arnold W. Lambisia, Zaydah R. de Laurent, Maureen W. Mburu, Edidah M. Ong’era, John M. Morobe, Edward Otieno, Hamza Abdou Azali, Kamal Said Abdallah, Abdoulaye Diarra, Ali Ahmed Yahaya, Peter Borus, Nicksy Gumede Moeletsi, Dratibi Fred Athanasius, Benjamin Tsofa, Philip Bejon, D. James Nokes, Lynette Isabella Ochola-Oyier

**Affiliations:** 1 Kenya Medical Research Institute (KEMRI) - Wellcome Trust Research Programme, Kilifi, Kenya, KILIFI, KILIFI, 230-80108, Kenya; 2 Institut National de Recherche pour l'Agriculture, la Pêche et l'Environnement (INRAPE), Comoros, Comoros; 3 Malaria National Referral Laboratory, Comoros; World Health Organisation (WHO) Country Office., Comoros, Comoros; 4 WHO/Africa Regional Office (AFRO), Brazzaville, Congo, Democratic Republic; 5 WHO Country Office, Nairobi, Kenya; 6 Nuffield Department of Medicine, Oxford University, United Kingdom (UK), Oxford, UK; 7 University of Warwick, Coventry, UK

**Keywords:** SARS-CoV-2, 501Y.V2, Comoros

## Abstract

**Background.** Genomic data is key in understanding the spread and evolution of SARS-CoV-2 pandemic and informing the design and evaluation of interventions. However, SARS-CoV-2 genomic data remains scarce across Africa, with no reports yet from the Indian Ocean islands.

**Methods.** We genome sequenced six SARS-CoV-2 positive samples from the first major infection wave in the Union of Comoros in January 2021 and undertook detailed phylogenetic analysis.

**Results.** All the recovered six genomes classified within the 501Y.V2 variant of concern (also known as lineage B.1.351) and appeared to be from 2 sub-clusters with the most recent common ancestor dated 30 ^th^ Oct-2020 (95% Credibility Interval: 06 ^th^ Sep-2020 to 10 ^th^ Dec-2020). Comparison of the Comoros genomes with those of 501Y.V2 variant of concern from other countries deposited into the GISAID database revealed their close association with viruses identified in France and Mayotte (part of the Comoros archipelago and a France, Overseas Department).

**Conclusions.** The recovered genomes, albeit few, confirmed local transmission following probably multiple introductions of the SARS-CoV-2 501Y.V2 variant of concern during the Comoros’s first major COVID-19 wave. These findings demonstrate the importance of genomic surveillance and have implications for ongoing control strategies on the islands.

## Introduction

Although Comoros, an island country in the Indian ocean, detected its first case of SARS-CoV-2 on 30 ^th^ April 2020, it experienced its first major SARS-CoV-2 outbreak in January 2021 i.e., 10 months later ^1^. By 28 ^th^ February 2021, Comoros had 3,571 laboratory-confirmed SARS-CoV-2 infections, 2,748 (76.9%) of which were confirmed after 1 ^st^ January 2021.

Genomic surveillance has been key in understanding the introduction, spread, and evolution of SARS-CoV-2 pandemic into countries since its emergence in late 2019 in China and in informing the design and evaluation of interventions ^2– 4^. Towards the end of 2020, in widely different geographical locations globally, three SARS-CoV-2 variants of concern (Alpha, Beta, and Gamma) emerged that appeared to be considerably more transmissible and with potential to facilitate immune escape or cause more severe disease than the prior SARS-CoV-2 variants ^5– 7^. The three variants possessed several defining amino acid changes, most of them occurring within the immunogenic spike (S) protein ^8^. The S protein contains the domain that binds the virus to the human host cell receptor and is a key target for several vaccines ^9^. Here, we investigated if the variants of concern had a role in the rising number of SARS-CoV-2 cases in the Union of Comoros in January 2021.

## Methods

An earlier version of this article can be found on bioRxiv (DOI: https://doi.org/10.1101/2021.04.08.21254321).

### Ethical statement

The SARS-CoV-2 genomes were generated as part of a regional collaborative COVID-19 public health rapid response. The whole genome sequencing study protocol was reviewed and approved by the Scientific and Ethics Review Committee (SERU), Kenya Medical Research Institute (KEMRI), Kenya (SERU #4035). Individual patient consent was not required by the committee for the use of these samples for sequencing as a part of the public health emergency response.

### Study site and samples

The samples analysed had been collected between 5 ^th^ and 11 ^th^ January 2021 in the Union of Comoros, specifically from two islands, Ngazidja and Mohéli ( Table 1). A total of 11 positive nasopharyngeal/oropharyngeal swab samples were sent to KEMRI-Wellcome Trust Programme (KWTRP) in Kilifi, Kenya, for genome analysis. KWTRP is one of the 12 designated WHO-AFRO /Africa-CDC specialized and regional reference laboratories for SARS-CoV-2 sequencing in Africa ^10^. Sample size was determined by the sequence available from the public health response.

**Table 1.** Baseline characteristics of the samples that were submitted to KEMRI-Wellcome Trust for sequencing and sequencing success.

Study ID	GISAID Accession Number	Sex	Island	Date sampling	Ct N gene, ORF1ab	Genome length ^π^, %
**C001**	EPI_ISL_1323658	Male	Ngazidja	Jan-2021	24.06, 26.42	26960, 90%
**C002**	EPI_ISL_1323659	Female	Ngazidja	Jan-2021	23.8, 26.46	26638, 89%
**C003 ^#^**	-	Female	Ngazidja	-	35.64, undetermined	-
**C004 ^#^**	-	Female	Ngazidja	-	Undetermined, undetermined	-
**C005**	EPI_ISL_1323660	Female	Ngazidja	Jan-2021	21.4, 23.8	26640, 89%
**C006 ^¥, #^**	-	-		Jan-2021	29.98, 34.28	-
**C007**	EPI_ISL_1323661	Male	Mohéli	Jan-2021	24.21, 27.33	26358, 88%
**C008**	-	Male	Mohéli	Jan-2021	33.39, 35.79	-
**C009**	EPI_ISL_1323662	Female	Mohéli	Jan-2021	16.41, 18.59	26638, 89%
**C010**	-	Female	Mohéli	Jan-2021	31.8, 35.35	-
**C011**	EPI_ISL_1323663	Female	Mohéli	Jan-2021	18.77, 21.26	26400, 88%

^¥^Intensive care unit case. ^π^Complete SARS-CoV-2 genome is 29,903 nucleotides long (Wuhan 2019 reference, accession number: NC_045512.2). ^#^Experienced missing data/demographic details in the submitted forms and were not sequenced.

### Laboratory procedures

On receiving the samples on the 16 ^th^ and 17 ^th^ January 2021, viral RNA was extracted using the QIAamp Viral RNA Mini kit (52906, Qiagen, Hilden, Germany) following the manufacturer’s instructions and analysed using the Sansure Biotech Novel Coronavirus (2019-nCoV) Nucleic acid Diagnostic real-time RT-PCR commercial kit (S3102E, Sansure Inc., China) which targets the nucleocapsid (N) and ORF1ab regions. Nine of the 11 samples were confirmed as SARS-CoV-2 positive by both gene targets (cycle threshold (Ct) <38.0). We proceeded to sequence six samples that had a Ct value of ≤ 29.0. Samples with Ct above 29.0 were excluded because we observed in our laboratory that they frequently fail the downstream quality control steps before sequencing due to possession of low viral titres. The RNA was first reverse transcribed using the LunaScript® RT SuperMix Kit (E3010, New England Biolabs Inc., Germany) then amplified using the Q5® Hot Start High-Fidelity 2X Master Mix (NEB M0494; New England Biolabs Inc., Germany) along with the ARTIC nCoV-2019 version 3 primers ^11^. The resultant amplicons were taken forward for library preparation and MinION (Mk1B) (Oxford Nanopore Technology, Oxford) sequencing. The six samples were processed alongside 17 other samples from coastal Kenya to make a batch of 23 samples.

### Data analysis

The MinION ( https://nanoporetech.com/products/minion) sequencing read-outs (fast5 files) were base-called using the Guppy basecaller 4.4.0 fast model and subsequently demultiplexed. Consensus genomes assembled using a SARS-CoV-2 ARTIC Network Bioinformatics pipeline ^11^. A threshold of 20x coverage was required for a base to be included in the consensus genome otherwise it was masked to N. We retrieved 75 random Beta variant sequences from the GISAID database (selected by downloading all the Beta variant sequences that were available on 28 ^th^ February 2021 and then using an inhouse python script to sub-sample). These data were then aligned with the Union of Comoros genomes (now available on GISAID) using MAFFT v.7.313 ( https://mafft.cbrc.jp/alignment/software/). The alignment was manually inspected in SEAVIEW v 4.6.4 ( http://doua.prabi.fr/software/seaview) to spot any obvious misalignments. We reconstructed maximum likelihood (ML) phylogeny using IQTREE v.1.6.12 ( http://www.iqtree.org/). Branch support was evaluated using 1,000 bootstrap iterations. The presence of a molecular clock signal was inspected in TempEst v1.5.3 ( http://tree.bio.ed.ac.uk/software/tempest/). Linear regression of root-to-tip genetic distances against sampling dates were plotted in R v4.0.2 ( https://www.r-project.org/). We inferred time-scaled phylogenies in BEAST V1.10.4 ( https://beast.community/) under the HKY+G substitution model with an uncorrelated relaxed molecular clock assumption. BEAST Markov chain Monte Carlo (MCMC) runs were set to run 50 million steps with sampling after every 2,500 steps ^12^. The BEAST run output was analysed in Tracer v1.7.1 ( http://tree.bio.ed.ac.uk/software/tracer/). Dated maximum clade credibility phylogeny was inferred using TreeAnnotator v1.10.4 ( https://beast.community/treeannotator after discarding 10% as burn-in and visualized using FigTree v1.4.4 ( http://tree.bio.ed.ac.uk/software/figtree/).

## Results

We assembled >80% of the SARS-CoV-2 genome from each of the six sequenced samples ( Table 1). The recovered genomes were classified into the lineage B.1.351 using the Pangolin toolkit v2.3.0 ^13^. The genomes possessed six of the eight Beta variant defining amino acid changes in the S protein (i.e., L18F, D80A, D215G, K417N, D614G, and A701V) plus a known three amino acid deletion at positions 243–245. Two additional defining amino acid changes (E484K and N501K) which were unconfirmed fell within a region that was not sequenced due to PCR amplicon drop-off. Our findings and confirmation of the presence of the SARS-CoV-2 Beta variant in Comoros samples was conveyed to Union of Comoros authorities on the 22 ^nd^ January via the WHO-AFRO office to inform public health actions. 

**Figure 1.**
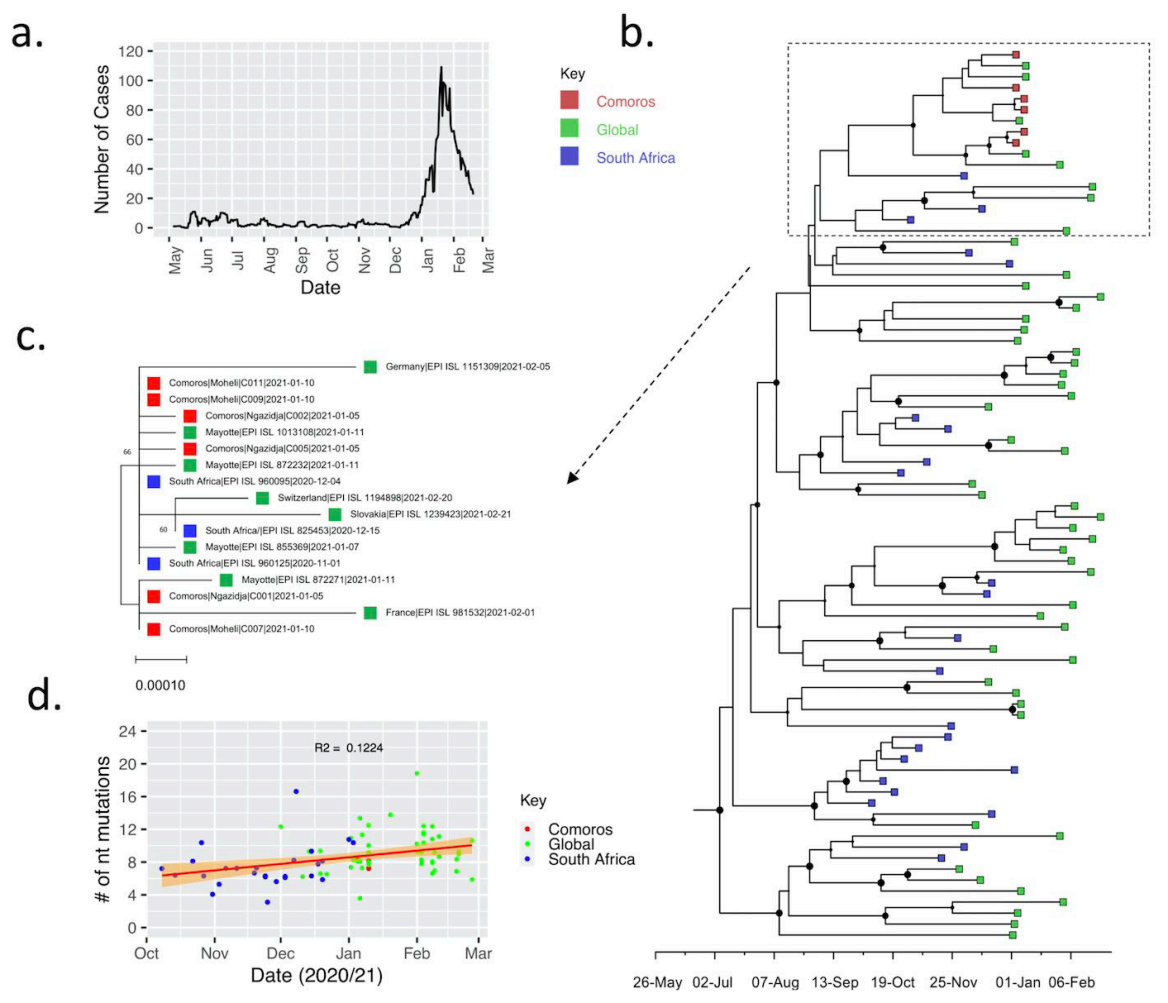
Genomic diversity of SARS-CoV-2 in Comoros Islands. **a**. Smoothed daily number of cases reported in Comoros Islands since the observation of the first case on 30 ^th^ April 2020 up to 19 ^th^ February 2020. **b**. time-scaled phylogenetic tree showing the placement of the six samples from Comoros (coloured in red) relative to other Beta variant sequences from around the globe coloured in green. Those from South Africa are coloured blue. **d**. a zoomed in Maximum Likelihood tree of the clade containing the newly sequenced Comoros genomes. **c**. Root-to-tip divergence among the analysed 81 Beta genomes.

The six Union of Comoros sequences differed only at three nucleotide positions: A13192G (1 genome), T23560C (2 genomes), and G27505T (1 genome). Compared to the Wuhan 2019 reference (Accession number: NC_45512.2), the Union of Comoros genomes had 21-22 nucleotide substitutions that translated into 16-17 amino acid changes. A time-scaled MCC phylogenetic tree of these sequences revealed that the Union of Comoros genomes formed a monophyletic group together with genomes from Mayotte (which is part of the Comoros archipelago and a French Overseas department) and France ( Figure 1). This group diverged into two sub-clusters with the most recent common ancestor dated 30 ^th^ Oct-2020 (95%CI: 06 ^th^ Sep-2020 to 10 ^th^ Dec-2020).

## Discussion

We provide evidence of circulation of the Beta variant during the first major SARS-CoV-2 epidemic peak in the Union of Comoros. The Beta variant was first identified in South Africa and had been reported in 41 countries as of 19 ^th^ February 2021. Initial data suggested that this variant exhibits up to 6-fold reduction in neutralization activity by post-vaccination sera or convalescent sera from individuals infected by prior variants ^14^. Thus, finding this variant in Union of Comoros is concerning since it has potential to overcome pre-existing immunity derived from natural infection or vaccination.

Understanding the extent of spread of this variant in Union Comoros is limited by the low number of cases sequenced. As of 28 ^th^ February 2021, the number of new cases in the Union of Comoros had considerably declined after peaking in mid-January ^1^. Comparison of the Union of Comoros genomes with genomes from across the globe found a close relation with those from the neighbouring Mayotte, a French Overseas Department. Mayotte detected its first case of SARS-CoV-2 on 10th March 2020 and experienced its first major SARS-CoV-2 outbreak from mid-January 2021 to mid-March 2021 thus overlapping with the Union of Comoros outbreak. 721 SARS-CoV-2 genomes were available on GISAID database from Mayotte as of 23 ^rd^ April 2021 and the majority (52%) were classified as Beta variant ^15^. Our study demonstrates how continental genomic surveillance using the Sequencing Laboratory Network for Covid-19 can be utilised to inform response to the SARS-CoV-2 pandemic.

## Data availability

### Underlying data

The six genomes have been deposited in GISAID ( https://www.gisaid.org/), Accession numbers EPI_ISL_1323658 - EPI_ISL_1323663 and can be accessed following registration and login. The Comoros daily case data for the period between April 2020 and February 2021 was obtained and is freely available from our in Our World in Data database ( https://ourworldindata.org/coronavirus/country/comoros).

Harvard Dataverse. Replication Data for: Detection of SARS-CoV-2 variant 501Y.V2 in Comoros Islands in January 2021. https://doi.org/10.7910/DVN/NDOIQ2.

This project contains the following underlying data:

- CAgoti_Comoros_Genome_Codebook.pdf. (Data table-1.tab structure description).
- CAgoti_Comoros_Genome_Readme.txt. (Readme file for data table-1.tab).
- DATA_Table-1.tab. (This dataset is part of the continuous genomic surveillance of SARS-CoV-2 courtesy of the Laboratory Sequencing Network created by WHO-AFRO and AFRICA-CDC. This dataset contains results of samples positive for SARS-CoV-2 by real-time PCR for the period 5th January 2020-10th January 2020).
- DATA_Table.tab. (This dataset is part of the continuous genomic surveillance of SARS-CoV-2 courtesy of the Laboratory Sequencing Network created by WHO-AFRO and AFRICA-CDC. This dataset contains results of samples positive for SARS-CoV-2 by real-time PCR for the period 5 ^th^ January 2020-10 ^th^ January 2020).
- gisaid_hcov-19_acknowledgement_table_2021_06_12_18.pdf. (This dataset acknowledges authors from the originating laboratories responsible for obtaining the specimens, as well as the submitting laboratories where the genome data were generated and shared via GISAID, on which this research is based).

Data are available under the terms of the Creative Commons Attribution 4.0 International license (CC-BY 4.0).
