# Supplementary material for: Detection of SARS-CoV-2 variant 501Y.V2 in Comoros Islands in January 2021
Source: Wellcome Open Res. 2021 Jul 28;6:192. [Version 1] doi: 10.12688/wellcomeopenres.16889.1 (PMC8753575; doi:10.12688/wellcomeopenres.16889.1)
Supplement: Peer review history

# Peer review: Reviewer response for version 1

**DOI:** 10.21956/wellcomeopenres.18631.r47738

**Published:** 2022-01-11

**Authors:** Aminu S. Jahun

**Affiliations:** 1 Division of Virology, Department of Pathology, University of Cambridge, Addenbrooke’s Hospital Level 5, Cambridge, CB2 0QQ, UK

Agoti *et al. *report on the sequencing and analyses of six SARS-CoV-2 samples from the Comoros Islands, all of which were found to be of the B.1.351 lineage. The manuscript is clear and concise, and their data will be of significant interest to officials in-charge of public health responses, and to the global scientific community.

 I only have a few minor comments as follows:

- I noticed that there was no mention of the inclusion of negative controls in the sequencing assays, and how the presence of mapped reads in the negative controls, if any, was addressed. This is particularly relevant given the high similarity between the six samples.
- Including the assembly pipeline version, and indicating which tool was used in generating the consensus sequence (nonopolish or medaka) may be useful for others who may be interested in including these data in future analyses
- In Table 1, perhaps just 'genome coverage', +/- number of Ns, may be more appropriate than 'genome length'.
- Figure 1 legend labels 'c' and 'd' are swapped (typo?).
- Also potential typo - 1st sentence, 4th paragraph on page 4 of the pdf, 'Union of Comoros' instead of 'Union Comoros'.

Is the work clearly and accurately presented and does it cite the current literature?

Yes

If applicable, is the statistical analysis and its interpretation appropriate?

Not applicable

Are all the source data underlying the results available to ensure full reproducibility?

Yes

Is the study design appropriate and is the work technically sound?

Yes

Are the conclusions drawn adequately supported by the results?

Yes

Are sufficient details of methods and analysis provided to allow replication by others?

Partly

Reviewer Expertise:

SARS-CoV-2 sequencing and analyses

I confirm that I have read this submission and believe that I have an appropriate level of expertise to confirm that it is of an acceptable scientific standard.

# Peer review: Reviewer response for version 1

**DOI:** 10.21956/wellcomeopenres.18631.r47089

**Published:** 2021-12-01

**Authors:** I-Ching Sam

**Affiliations:** 1 Department of Medical Microbiology, Faculty of Medicine, University of Malaya, Kuala Lumpur, Malaysia

This is a brief report based on 6 SARS-CoV-2 sequences from the Union of Comoros, which were the only Comoros sequences available in GISAID at the time of publication (July 2021). The samples were from January 2021, during the first major COVID-19 wave. Standard methods were used. Sequences were shown to be of the beta variant of concern and clustered together, similar to sequences from nearby Mayotte and South Africa. Although limited by the small number of sequences and lack of epidemiological data, the report is of local and regional interest as there is little/no molecular epidemiology data from Comoros.

 There are no major issues.

**Minor issues:**

- More relevant details of the setting may help increase understanding of the significance of the findings. For example, case incidence per 100,000 during this wave, incidence in Ngazidja and Mohéli islands, travel restrictions and likely sources of importation, etc.
- Is there any further epidemiological data for these 6 cases? e.g. travel history, contact with each other.
- Fig 1a legend has a typo, should be "19th Feb 2021".
- Fig 1 legend, (d) and (c) are the wrong way round.

Is the work clearly and accurately presented and does it cite the current literature?

Yes

If applicable, is the statistical analysis and its interpretation appropriate?

Yes

Are all the source data underlying the results available to ensure full reproducibility?

Yes

Is the study design appropriate and is the work technically sound?

Yes

Are the conclusions drawn adequately supported by the results?

Yes

Are sufficient details of methods and analysis provided to allow replication by others?

Yes

Reviewer Expertise:

Medical virology, respiratory viruses

I confirm that I have read this submission and believe that I have an appropriate level of expertise to confirm that it is of an acceptable scientific standard.
